# Substantia nigra alterations in mice modeling Parkinson’s disease

**DOI:** 10.18699/vjgb-24-82

**Published:** 2024-11

**Authors:** I.N. Rozhkova, S.V. Okotrub, E.Yu. Brusentsev, T.A. Rakhmanova, D.A. Lebedeva, V.S. Kozeneva, N.A. Shavshaeva, N.V. Khotskin, S.Ya. Amstislavsky

**Affiliations:** Institute of Cytology and Genetics of the Siberian Branch of the Russian Academy of Sciences, Novosibirsk, Russia; Institute of Cytology and Genetics of the Siberian Branch of the Russian Academy of Sciences, Novosibirsk, Russia; Institute of Cytology and Genetics of the Siberian Branch of the Russian Academy of Sciences, Novosibirsk, Russia; Institute of Cytology and Genetics of the Siberian Branch of the Russian Academy of Sciences, Novosibirsk, Russia Novosibirsk State University, Novosibirsk, Russia; Institute of Cytology and Genetics of the Siberian Branch of the Russian Academy of Sciences, Novosibirsk, Russia; Institute of Cytology and Genetics of the Siberian Branch of the Russian Academy of Sciences, Novosibirsk, Russia Novosibirsk State University, Novosibirsk, Russia; Institute of Cytology and Genetics of the Siberian Branch of the Russian Academy of Sciences, Novosibirsk, Russia Novosibirsk State University, Novosibirsk, Russia; Institute of Cytology and Genetics of the Siberian Branch of the Russian Academy of Sciences, Novosibirsk, Russia; Institute of Cytology and Genetics of the Siberian Branch of the Russian Academy of Sciences, Novosibirsk, Russia

**Keywords:** mice, Parkinson’s disease, motor coordination, dopaminergic brain system, alpha-synuclein, мыши, болезнь Паркинсона, координация движений, дофаминергическая система мозга, альфа-синуклеин

## Abstract

Parkinson’s disease (PD) is an age-related neurodegenerative pathology of the central nervous system. The well-known abnormalities characteristic of PD are dysfunctions in the nigrostriatal system including the substantia nigra of the midbrain and the striatum. Moreover, in PD persons, alpha-synucleinopathy is associated with abnormalities in the dopaminergic brain system. To study the mechanisms of this pathology, genetic models in mice have been designed. Transgenic mice of the B6.Cg-Tg(Prnp-SNCA*A53T)23Mkle/J strain (referred to as B6.Cg-Tg further in the text) possess the A53T mutation in the human alpha-synuclein SNCA gene. The density of neurons in the prefrontal cortex, hippocampus, substantia nigra and striatum in B6.Cg-Tg mice was assessed in our previous work, but the dopaminergic system was not studied there, although it plays a key role in the development of PD. The aim of the current study was to investigate motor coordination and body balance, as well as dopaminergic neuronal density and alpha-synuclein accumulation in the substantia nigra in male B6.Cg-Tg mice at the age of six months. Wild-type mice of the same sex and age, siblings of the B6.Cg-Tg mice from the same litters, lacking the SNCA gene with the A53T mutation, but expressing murine alpha-synuclein, were used as controls (referred to as the wild type further in the text). Motor coordination and body balance were assessed with the rota-rod test; the density of dopaminergic neurons and accumulation of alpha-synuclein in the substantia nigra were evaluated by the immunohistochemical method. There was no difference between B6.Cg-Tg mice and WT siblings in motor coordination and body balance. However, accumulation of alpha-synuclein and a decrease in the number of dopaminergic neurons in the substantia nigra were found in the B6.Cg-Tg mouse strain. Thus, the mice of the B6.Cg-Tg strain at the age of six months have some symptoms of the onset of PD, such as the accumulation of mutant alpha-synuclein and a decrease in the number of dopaminergic neurons in the substantia nigra. Taken together, the results obtained in our work qualify the B6.Cg-Tg strain as a pertinent model for studying the early stage of human PD already at the age of six months.

## Abstract (ru)

Болезнь Паркинсона (БП) – возрастная нейродегенеративная патология центральной нервной системы. Наиболее характерными нарушениями при БП считаются аномалии в нигростриарной системе, включающей в себя черную субстанцию среднего мозга и полосатое тело. При БП аномалии дофаминергической системы мозга сопровождаются альфа-синуклеинопатией. Для изучения механизмов возникновения данной патологии созданы генетические модели на мышах. Трансгенные мыши линии B6.Cg-Tg(PrNp-SNCA*A53T)23Mkle/J (далее по тексту B6.Cg-Tg) имеют мутацию A53T в гене SNCA альфа-синуклеина человека. В нашей предыдущей работе оценена плотность нейронов в префронтальной коре, гиппокампе, черной субстанции и полосатом теле у мышей этой линии, однако дофаминергическая система мозга, которая играет ключевую роль в развитии БП, изучена не была. Целью настоящего исследования стало изучение координации движений и баланса тела, а также плотности дофаминовых нейронов и накопления альфа-синуклеина в черной субстанции самцов мышей линии B6.Cg-Tg в возрасте шести месяцев. В качестве контроля использованы сибсы, у которых не было экспрессии гена SNCA с мутацией A53T и экспрессировался мышиный альфа-синуклеин (далее по тексту – дикий тип; wild type, WT), того же пола и возраста, из тех же самых выводков, что и исследуемые мыши B6.Cg-Tg. Координация движений и баланс тела были изучены с помощью теста «рота-род»; плотность дофаминовых нейронов и накопление альфа-синуклеина в черной субстанции оценены иммуногистохимическим методом. Полученные результаты показывают, что мыши B6.Cg-Tg не имеют отличий по координации движений и баланса тела от контроля – сибсов дикого типа. Однако у мышей B6.Cg-Tg в черной субстанции были обнаружены накопление альфа-синуклеина и уменьшение числа дофаминовых нейронов. Таким образом, мыши линии B6.Cg-Tg в возрасте шести месяцев имеют симптомы начала развития БП, такие как накопление мутантного альфа-синуклеина и уменьшение числа дофаминовых нейронов в черной субстанции. Полученные в этом исследовании результаты позволяют характеризовать линию B6.Cg-Tg в качестве адекватной модели для изучения ранней стадии БП человека уже в возрасте шести месяцев

## Introduction

Parkinson’s disease (PD) is the second most common neurodegenerative disorder in humans. It manifests primarily in idiopathic and sporadic forms (Beitz, 2014; Tran et al., 2020; Bidesi et al., 2021). PD’s main symptoms include impaired motor function, muscle rigidity, resting tremors, and bradykinesia (Halliday et al., 2006; Beitz, 2014). Non-motor symptoms such as sleep disturbances, neuropsychiatric conditions, and cognitive deficits are also prevalent (Beitz, 2014; Hayes, 2019). Age-related changes in various brain areas are common in PD (Halliday et al., 2006; Dickson et al., 2009).

This pathology is characterized by a variety of abnormalities related to the synthesis of neurotransmitters in the brain and thefunctioning of their receptors (Jellinger, 1991; Deutch et al., 2006). The most common brain pathology associated with PD involves the nigrostriatal pathway, which encompasses the substantia nigra (SNC) in the midbrain and the striatum (STR) (Dickson et al., 2009; Beitz, 2014; Hayes, 2019). This pathway is crucial for motor control, as it activates dopaminergic neurons that regulate flexor and extensor muscle movements (Korchounov et al., 2010).

Another key feature of PD is the accumulation of alphasynuclein in brain neurons, particularly in the SNC and STR (Dickson et al., 2009; Burre et al., 2018; Lai et al., 2021). Alpha-synuclein regulates synaptic activity, including dopamine synthesis, transport, and storage (Burre et al., 2018; Bidesi et al., 2021). Mutations in the alpha-synuclein gene, such as A53T and A30P, result in abnormal protein folding and aggregation (Polymeropoulos et al., 1997; Spillantini et al., 1997). These changes lead to neuronal loss and alpha-synuclein accumulation, both hallmark features of PD (Dickson et al., 2009; Venda et al., 2010; Poewe et al., 2017).

Animal models are used to study PD mechanisms and identify potential treatment strategies. These models are typically classified as toxic or genetically engineered (Grigoryan, Bazyan, 2007; Korolenko et al., 2020). Studies have shown that damage to neurons in the STR leads to simultaneous flexor and extensor muscle impairment (Stern, 1966; Kato, Kimura, 1992). In PD models, motor deficits are often accompanied by alpha-synucleinopathy (Dickson, 2018).

Commonly used PD models include transgenic mice expressing mutant forms of the human alpha-synuclein (SNCA) gene, such as the A30P and A53T mutations (Unger et al., 2006; Grigoryan, Bazyan, 2007; Korolenko et al., 2020). These models display motor impairments that correlate with nigrostriatal degeneration (Chia et al., 2020). Transgenic mice with the A30P mutation in the SNCA gene typically exhibit a milder form of BP phenotype compared to mice with the A53T mutation, which makes it challenging to study behavioral characteristics and disorders in different brain structures (Van der Putten et al., 2000; Lee et al., 2001; Crabtree, Zhang, 2012). However, results can vary based on the genetic background, affecting the extent to which PD traits are expressed, how and under which promoter the gene was transferred, and on other factors (Crabtree, Zhang, 2012).

Various techniques are employed to investigate PD in mouse models (Graham, Sidhu, 2010; Tikhonova et al., 2020; Langley et al., 2021). In particular, the rotarod test (RR) is widely used to assess coordination of movement and body balance (Graham, Sidhu, 2010; Seo et al., 2020). However, the results of testing for the key features characterizing PD may vary if the same transgene is expressed on a different genetic background. The review article by D.M. Crabtree and J. Zhang (2012) describes in detail how different genetic factors affect the manifestation of the pathology emphasizing the role of genetic background on which the transgenic model was designed and the promoter under which the transgene was embedded. The study of Paumier et al. (2013) indicates that transgenic mice that express human alpha-synuclein with the A53T mutation at the age of two months exhibit a longer latency time before the drop from a rotating rod during the rotarod test (which evaluates coordination of movement and body balance) compared to the wild-type siblings. Results of other studies exploiting the transgenic mouse strains with the same A53T mutation were either opposite (Graham, Sidhu, 2010) or demonstrated no differences in the RR test results between the wild-type control animals and the transgenic ones even at the age of nine months (Liu et al., 2018) depending on the genetic background on which the transgenic mouse strain was obtained.

When studying mice modeling PD, immunohistochemical methods are also used to study various brain structures (Tang et al., 2017; Korolenko et al., 2020; Langley et al., 2021). The focus of these studies is on the SNC and STR, which are significantly altered in PD (Burre et al., 2018; Lai et al., 2021).

Transgenic hemizygous mice of the B6.Cg-Tg(PrNp- SNCA*A53T)23Mkle/J strain (hereinafter referred to as B6.CgTg), which express the A53T mutation in the human alpha-synuclein SNCA gene, were first produced at the Jackson Laboratory (USA) (https://www.jax.org/strain/006823). This gene is not expressed in all individuals (Unger et al., 2006), and therefore, among siblings, there may be transgenic as well as wild-type mice. The model replicates the behavioral features and reproduces the symptoms of synucleinopathy, specifically, the age-related neurodegenerative changes characteristic of PD (Pupyshev et al., 2018; Korolenko et al., 2020; Seo et al., 2020; Zhang et al., 2022).

In studies of B6.Cg-Tg mice as PD models, C57BL/6J mice are almost always selected and used as a control group (Pupyshev et al., 2018; Seo et al., 2020). The maternal environment has a significant impact on offspring, mediated through epigenetic processes, both during the gestation and the early postnatal period (Case et al., 2010; Nicholas, Ozanne, 2019; Wu, Dean, 2020). Selection of an appropriate control group is an essential part of the experimental design as it allows to avoid variables that may influence the estimated parameters characterizing PD. In particular, maternal factors may affect prenatal (Wu, Dean, 2020) and early postnatal development, especially during the weaning period (Case et al., 2010). To minimize the maternal influence, it was recommended to compare siblings, especially in the studies involving transgenic and knockout animals (Holmdahl, Malissen, 2012; Chen et al., 2020). Thus, the study conducted on mice of the B6.Cg-Tg strain needs wild-type siblings as controls.

The nigrostriatal pathway of the brain, which has been implicated in the development of Parkinson’s disease, has not been sufficiently explored in B6.Cg-Tg mice that model human PD with the A53T mutation in the SNCA gene. In our previous study (Rozhkova et al., 2023), we investigated the density of neurons in the prefrontal cortex, hippocampus, SNC, and STR of B6.Cg-Tg mice at an early stage of this pathology, specifically at the age of six months. However, the dopaminergic system, which is crucial for the development of PD, was not examined.

The aim of this work was to compare male mice of the B6.Cg-Tg strain with wild-type (WT) siblings by the following parameters: 1) coordination of movements and body balance; 2) accumulation of alpha-synuclein; and 3) the density of dopamine neurons in the substantia nigra.

## Materials and methods

Experimental animals. Male siblings resulting from mating C57BL/6J females (4) with hemizygous B6.Cg-Tg males (4) were used in this experiment. In total, four litters were obtained, and genotyping of the offspring was performed. The experimental group consisted of animals carrying the A53T mutation (B6.Cg-Tg), while the remaining animals served as wild-type (WT) controls. Five hemizygous B6.Cg-Tg males and 10 WT males, which lacked the A53T mutation in the SNCA gene, were used.

All animals were housed in the SPF-vivarium at the Institute of Cytology and Genetics SB RAS (Novosibirsk, Russia) in individually ventilated OptiMice cages (Animal Care, USA), measuring 34.3 × 29.2 × 15.5 cm. They were kept at 22–24 °C with a humidity level of 40–50 % and an inverted 12:12-hour day-night cycle (sunrise at 3 a. m.). Fractionated birch chips (ТU 16.10.23-001-0084157135-2019) were used as bedding. The animals had free access to standardized chew (“Delta Feeds” LbK 120 R-22, GOST 34566-2019, BioPro, Russia) and purified water (“Severjanka”, Ecoproject, Russia).

All experiments were approved by the Bioethics Committee of the Institute of Cytology and Genetics SB RAS (protocol No. 145, March 29, 2023) and complied with the European Convention for the Protection of Vertebrate Animals used for Experimental and Other Scientific Purposes.

Study of motor coordination and body balance. At six months of age, five male B6.Cg-Tg mice with the A53T mutation and 10 wild-type controls from the same litters were tested. Motor coordination and body balance were assessed using the rotarod (RR) test. Two days before testing, the animals were isolated in clean individual OptiMice cages (34.3 × 29.2 × 15.5 cm). All equipment was sanitized using a 6 % hydrogen peroxide solution before testing.

The accelerated RR test is widely used to assess movement coordination and balance, particularly in studies of neurodegenerative conditions such as PD (Seo et al., 2020). The Ugo Basile 47600 device (Ugo Basile, Italy) consists of five 5.7 cm-wide tracks with 3 cm drums at a height of 16 cm, separated by flat round parts. The device has dimensions of 27.94 × 43.18 × 38.10 cm, weighs 6.4 kg, and accelerates between 2–80 rpm. The rotarod device was programmed to increase speed linearly from 5 to 40 rpm over 300 seconds. Three sessions were conducted for each mouse, with oneminute breaks between sessions. The time taken for the mouse to fall off the rod was recorded for each run. After the RR test, the brains of five B6.Cg-Tg mice (confirmed for the A53T mutation) and five wild-type controls from the same litters were studied. Animals were randomly selected from four litters, and brain analysis was conducted following intracardiac perfusion, as described below.

Intracardiac perfusion. Brain tissue fixation was performed following the method described by Rozhkova et al. (2023). Mice were anesthetized with intraperitoneal injections of medetomidine hydrochloride (Meditin, 0.01 mg/ kg; Api-San, Russia) and zoletil (Zoletil, 50 mg/kg; Virbac, France) 10 minutes later. Perfusion was carried out using 15 ml of phosphate-buffered saline (PBS), followed by 15 mL of 10 % formalin. The brain was removed and placed in a 30 % sucrose solution based on PBS with 5 mL of 10 % formalin for dehydration and fixation. After two weeks being kept at +4 °C, the tissue samples sank to the bottom of the tube. The samples were then embedded in O.C.T Tissue-Tek (Sakura Finetek, USA) and frozen at –70 °C in an MDF-594 horizontal low-temperature freezer (Sanyo, Japan).

Preparation of frozen brain sections. Brain sections from the substantia nigra (SNC) were prepared at a distance of –3.08 to –3.52 mm from bregma, following the G. Paxinos and K. Franklin atlas (Paxinos, Franklin, 2012). Frozen sections, 10 μm thick, were prepared using an HM550 OP Cryotome (Thermo Scientific, USA) at –25 °C and placed on PCI-coated adhesive glass slides with polished edges (CITOTEST, China).

Immunohistochemical analysis. The samples were stained according to the antibody kit manufacturers’ protocols. Before staining, sections were dehydrated and then rehydrated for five minutes in PBS. Heat-induced antigen retrieval was performed using 10 mM alkaline citrate buffer (pH 9) at 95 °C for 15 minutes in a TW-2.02 water bath (Elmi, Latvia). Thereafter sections were removed from the buffer and cooled to room temperature. Samples were washed three times in PBS-Tween buffer: PBS supplemented with 0.1 % Tween-20 (P9416-100 mL; Sigma-Aldrich, USA) for 15 min. Each section was then covered with Protein Block buffer (ab64226; Abcam, UK) for 30 min, followed by the excess liquid removal according to the manufacturer’s recommendations.

After washing procedure and exposure to Protein Block buffer, 50 μL of primary antibody were added; the samples were left overnight at 4 °C in a humidified dark chamber. The concentrations of antibodies used were 1:450 for anti- Tyrosine Hydroxylase (TH) – anti-TH (ab6211; Abcam, UK). For alpha-synuclein detection, 50 μL of alpha-Synuclein Antibody primary antibody (NB110-61645, dilution, Novus Biologicals, Littleton, CO, USA) were added at a concentration of 1:600 and left for 36 h at 4 °C in a humidified dark chamber. The sections were then washed in PBS-Tween buffer for 15 min, excess liquid was removed, 50 μL of the secondary antibody Goat Anti-Rabbit IgG H&L AF488 (ab150077; Abcam, UK) were added at a concentration of 1:700. The samples were left in a humidified dark chamber for two hours at 4 °C. Thereafter, the samples were washed in PBS-Tween buffer for 15 min, excess liquid was removed, and the samples were placed in ProLong, Glass Antifade Mountant medium (Thermo P36982; Thermo Fisher Scientific, USA). When assessing alpha-synuclein in neurons, in order to identify neurons, 80 μL of DAPI (Maric et al., 2021) were additionally added to the sections for 15 min and then washed twice with PBS for 3 min. After adding antibodies, the sections were placed in a humidified dark chamber.

Neuronal density analysis. The density of antibodylabeled neurons was assessed using an Axio Imager.M2 microscope (Carl Zeiss, Germany) equipped with a Zeiss Axiocam 506 mono camera. Neuronal counts were performed using ImageJ software (National Institutes of Health, USA), and neuronal density was calculated as the number of neurons per cubic millimeter (mm3), as described in Rozhkova et al. (2023).

Statistical analysis. Data were analyzed using STATISTICA v. 12.0 software (StatSoft, Inc., USA). Results are presented as medians (Me) with the first (q1) and third (q3) quartiles – Me [Q1;Q3]. Behavioral data (the median of three sessions) and neuron density were compared using the Mann–Whitney U-test. Statistical significance was set at p < 0.05.

## Results

**Fig. 1.**
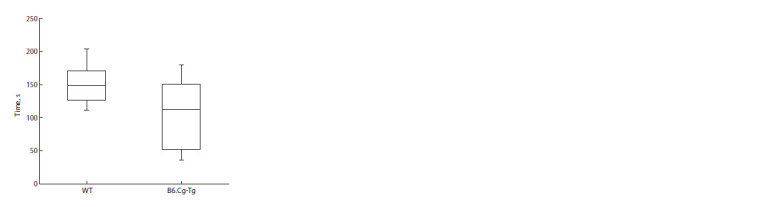
Latency before the drop in the rotarod test for male offspring of B6.Cg-Tg mice and their wild-type siblings at the age of six months.

**Fig. 2.**
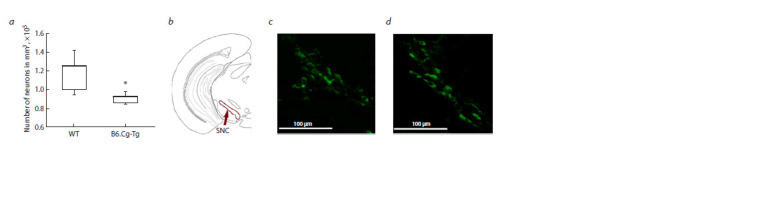
Density of dopaminergic neurons in the substantia nigra (SNC), the neurons were labeled with antibodies against tyrosine hydroxylase – TH. a – number of neurons per mm3; b – schematic representation of the region of interest in the brain. Microphotographs of the sections in this region: c – wild type (WT); d – B6.Cg-Tg. * p < 0.05.

**Fig. 3.**
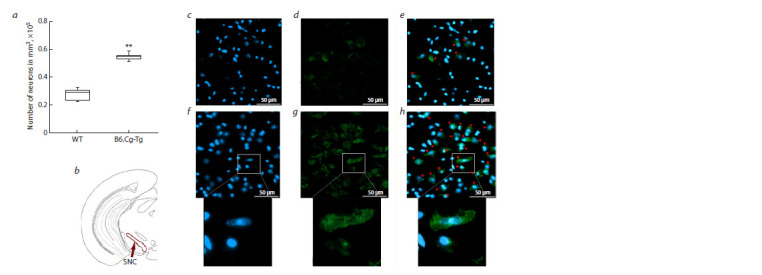
Density of neurons with alpha-synuclein in the substantia nigra (SNC), neurons are labeled with antibodies against alpha-synuclein, neuronal nuclei are stained with DAPI. a – number of neurons per mm3; b – schematic representation of the region of interest in the brain. Microphotographs of the sections in this region: c–e – wild type, f–h – B6.Cg-Tg; c, f – DAPI; d, g – alpha-synuclein; e, h – merged images. Arrowheads indicate neurons with alpha-synuclein inclusions. Figures in the white boxes represent high-magnification images. ** p < 0.01.

Data from the RR test are presented in Figure 1. Statistical analysis using the Mann–Whitney test showed no significant differences in latency between B6.Cg-Tg mice and wild-type controls. Neuronal density data for TH-labeled neurons in the substantia nigra are shown in Figure 2. The analysis revealed a significantly lower density of dopamine neurons in B6.Cg-Tg mice compared to wild-type controls ( p < 0.05), with 0.92 × 105 [0.86 × 105; 0.93 × 105] versus 1.25 × 105 [1.00 × 105; 1.26 × 105] neurons, respectively. Data on the density of neurons labeled for alpha-synuclein in the substantia nigra are shown in Figure 3. Statistical analysis indicated a significant increase in alpha-synuclein-positive neurons in B6.Cg-Tg mice compared to wild-type controls ( p < 0.01), with 0.55 × 105 [0.53 × 105; 0.56 × 105] neurons versus 0.29 × 105 [0.23 × 105; 0.31 × 105].

## Discussion

The rotarod test is widely used to evaluate motor activity and function in mice with neurodegenerative disorders (Graham, Sidhu, 2010; Oaks et al., 2013; Seo et al., 2020). In mice with the A53T mutation, motor activity changes typically begin between 2 and 12 months of age. This is linked to the synthesis of alpha-synuclein in brain neurons (Unger et al., 2006; Graham, Sidhu, 2010; Wang et al., 2022). Therefore, behavioral testing during this time is suitable (Zhang et al., 2019). In one study, A53T-a-Syn mice of various ages underwent nine rotarod sessions over three days, and the average results were compared (Oaks et al., 2013). The findings revealed worse motor coordination in A53T-a-Syn mice than in control animals as early as two and four months old (Oaks et al., 2013).

Similar results were seen in two-month-old mice from other transgenic lines carrying the SNCA gene and the A53T mutation, also using the rotarod test (Zhang et al., 2019). However, in older mice, these motor differences leveled out or were even reversed (Graham, Sidhu, 2010). In this study, as well as in the J.H. Seo et al. (2020) report, no differences in coordination and balance were found between six-month-old B6.Cg-Tg mice and wild-type littermates in the accelerated rotarod test. Depending on the Parkinson’s disease (PD) model, motor impairments can emerge either earlier (Oaks et al., 2013; Zhang et al., 2019) or later (Graham, Sidhu, 2010; Seo et al., 2020). In our results, early-stage B6.Cg- Tg mice did not show motor deficits, similar to human PD where motor symptoms often arise later in life as the disease progresses (Halliday et al., 2006).

It is well established that Parkinson’s disease causes disruptions in the nigrostriatal pathway (Dickson et al., 2009; Beitz, 2014; Hayes, 2019). Damage to these brain structures serves as a key marker of PD in both humans and animal models (Kato, Kimura, 1992; Unger et al., 2006; Beitz, 2014; Taguchi et al., 2020). Our previous research showed a reduction in the number of neurons in the substantia nigra of male B6.Cg-Tg mice (Rozhkova et al., 2023). In this study, we also observed fewer dopaminergic neurons in this region compared to wild-type mice. Similar results were reported in BAC-SNCAA53T/– mice, where a reduction in TH-positive neurons in the substantia nigra was observed (Taguchi et al., 2020). Additionally, A53T-Tg mice showed a reduced response to therapeutic dopamine treatments (Unger et al., 2006). Dopaminergic neurons in the substantia nigra are critical for regulating motor activity (Schultz et al., 1983). Despite this, our six-month-old B6.Cg-Tg mice did not exhibit motor deficits, although these may develop later (Chia et al., 2020).

The current study also found more alpha-synuclein-positive neurons in the substantia nigra of B6.Cg-Tg mice than in WT littermates, which mirrors findings in human PD cases (Dickson, 2018; Bae et al., 2021). This is a common trait in various mouse models of PD (Vander Putten et al., 2000; Taguchi et al., 2020; Wang et al., 2022). A similar result was found in certain transgenic SNCA mouse strains with the A53T mutation, where alpha-synuclein oligomers increased in the substantia nigra starting at three months old (Taguchi et al., 2020; Wang et al., 2022). In Pitx3- A53T-a-Syn mice aged six to 18 months, alpha-synuclein accumulation in the substantia nigra increased compared to C57BL controls. This was linked to significant degeneration of parvalbumin-positive neurons (Zheng et al., 2022). The connection between alpha-synuclein inclusions and neuron death is well established (Kalia et al., 2013).

Toxic alpha-synuclein oligomers can disrupt protein expression and endoplasmic reticulum function (Kalia et al., 2013). Mutant alpha-synuclein buildup in brain neurons is likely key to PD symptoms. Both the A53T and A30P mutations cause alpha-synuclein to become less soluble (Grigoryan, Bazyan, 2007). Our findings show that an increase in alpha-synuclein-positive neurons in the substantia nigra of B6.Cg-Tg mice is already evident by six months of age.

## Conclusion

In this study, we characterized six-month-old B6.Cg-Tg (PrNp-SNCA*A53T)23Mkle/J mice for markers important for Parkinson’s disease manifestation, such as motor coordination and body balance, as well as the density of dopamine neurons and alpha-synuclein neurons in the substantia nigra. Six-month-old B6.Cg-Tg mice show early signs of Parkinson’s disease. These include the accumulation of mutant alpha-synuclein and a reduced number of dopaminergic neurons in the substantia nigra. Our results suggest that these mice can serve as a suitable model for studying the early stages of Parkinson’s disease in humans. This model offers valuable insights for future research on disease onset and potential treatments.

## Conflict of interest

The authors declare no conflict of interest.
